# Adapting Child Health Knowledge Translation Tools for Use by Indigenous Communities: Qualitative Study Exploring Health Care Providers’ Perspectives

**DOI:** 10.2196/36353

**Published:** 2022-10-05

**Authors:** Sarah A Elliott, Jason Kreutz, Kelsey S Wright, Sherri Di Lallo, Shannon D Scott, Lisa Hartling

**Affiliations:** 1 Alberta Research Centre for Health Evidence, Department of Pediatrics Faculty of Medicine and Dentistry University of Alberta Edmonton, AB Canada; 2 Cochrane Child Health University of Alberta Edmonton, AB Canada; 3 Stollery Awasisak Indigenous Health Stollery Children's Hospital Edmonton, AB Canada; 4 Faculty of Nursing University of Alberta Edmonton, AB Canada

**Keywords:** knowledge translation, culture, Indigenous culture, child health, adaptation

## Abstract

**Background:**

Our research groups have developed a number of parental knowledge translation (KT) tools to help families understand common childhood illnesses and make informed decisions regarding when to seek urgent care. We have developed a series of videos to help parents understand how to manage common acute childhood illnesses at home and when to contact emergency health care services. It is unclear whether the videos in their current form and language are useful for a wider range of populations, including Indigenous groups.

**Objective:**

The purpose of this study was to explore whether and understand how our KT tools could be adapted for use with Indigenous communities.

**Methods:**

Health care providers (HCPs) serving Indigenous families in Alberta, Canada, were asked to review 2 of our KT tools (one on croup and one on acute otitis media), complete a demographic survey, and participate in a one-on-one semistructured interview. HCPs were asked to reflect on the usability of the KT tools within their practice and what cultural adaptation considerations they felt would be needed to develop KT tools that meet the needs of Indigenous clients. Audio recordings from the interviews were transcribed verbatim and analyzed for relevant themes using thematic analysis.

**Results:**

A total of 18 HCPs (n=15, 83% women and n=3, 17% men) from various health professions (eg, physician, registered nurse, and licensed practical nurse) were interviewed. Of these 18 HCPs, 7 (39%) self-identified as Indigenous. Four overarching themes were identified as important when considering how to adapt KT tools for use by Indigenous communities: accessibility, relatability, KT design, and relationship building. Access to tangible resources and personal and professional connections were considered important. Accessibility affects the types of KT tools that can be obtained or used by various individuals and communities and the extent to which they can implement recommendations given in those KT tools. In addition, the extent to which users relate to the depictions and content within KT tools must be considered. The environments, portrayals of characters, and cultural norms and values presented within KT tools should be relevant to users to increase the relatability and uptake of recommendations. Most importantly, fostering genuine and sustainable relationships with users and communities is a vital consideration for KT tool developers.

**Conclusions:**

These findings serve to cultivate a greater understanding of the various components that HCPs consider important when developing or culturally adapting KT tools for use by Indigenous families. This information will help support the effective adaptation and distribution of KT tools for use by a broad audience. Careful consideration of the themes identified in this study highlights the importance of working together with the knowledge users (health care consumers) when developing KT tools.

## Introduction

### Background

Effective synthesis, dissemination, and implementation of health research findings is critical for informing patient decision-making, promoting the effective use of health care system resources, and, ultimately, improving health outcomes [1]. Over the past few years, this process known as knowledge translation (KT) has been increasingly directed toward those accessing health care (ie, patients and parents) to foster an active involvement in informed decision-making [2]. Our research group strives to improve health outcomes for children by facilitating the uptake of evidence by actively engaging health care providers (HCPs) and families in the codevelopment and dissemination of a variety of user-friendly KT tools. We integrate the best available evidence (systematic reviews [3]) with parent experiences (qualitative interviews [4]) to create a successful KT model centered within the knowledge-to-action [5] cycle. We are now exploring within local contexts how best to tailor our KT products to diverse audiences.

Our research team has developed a series of web-based videos to help parents understand how to manage common acute childhood illnesses at home and when to contact health care services. These videos have been reviewed by our pediatric parent advisory group [6]; vetted by multidisciplinary, pediatric emergency health care professionals; tested for usability by parents in remote, rural, and urban settings [7,8]; and released on the web for the English-speaking general population (freely accessible at ECHO research [9]). However, it is unclear whether these videos, in their current form, will be useful for a wider range of population.

The relatability and accessibility of KT tools require developers to understand the contexts and realities in which their viewers find themselves [5,10]. Although KT tools are meant to present health care information in user-friendly and relatable formats, they are often developed with the majority cultural communities in mind (eg, in North America, this is predominantly White, English-speaking populations). Culturally adapting KT tools for use by diverse groups offers a great potential for decreasing health disparities and increasing access to resources.

In Canada, Indigenous populations face a plethora of health disparities and unique barriers when accessing health information [11,12]. This can include a lack of access to health resources and the discontinuity of care for families, which can directly affect child health outcomes [13,14]. Unfortunately, most existing KT initiatives may not effectively engage Indigenous populations or incorporate Indigenous knowledge and practices [15-17]. In addition, there is a lack of information in the literature on how best to implement, practice, and appraise KT initiatives for Indigenous populations [18].

### Objective

We sought to understand whether and how our KT tools could be adapted for use by Indigenous communities. Although frameworks to achieve cultural adaptation for health promotion programs and health interventions have been documented [19,20], guidance on how best to adapt KT products for use by culturally and linguistically diverse audiences is lacking. As the first step, we explored the opinions of HCPs who work with Indigenous communities regarding the applicability of 2 of our KT tools for use by the families they serve. We aimed to gather their perspectives on the usefulness and appropriateness of the tools as well as considerations for how best to adapt or develop KT tools in such a way that they are relatable and accessible to Indigenous communities.

## Methods

### Stakeholder Engagement

Within our province, there are 45 First Nations in 3 treaty areas, representing culturally distinct and traditionally underserved Indigenous communities [21]. We engaged with several local stakeholders (researchers, clinicians, program managers, and Indigenous community members) working in Indigenous health and research to understand what approach would work best. Those early conversations helped develop the research plan and facilitated subsequent engagement with HCPs who serve Indigenous families and communities in Alberta, Canada.

### Overview

Semistructured one-on-one web-based interviews were conducted with HCPs who serve Indigenous communities to discuss 2 existing child health KT videos (on croup and acute otitis media [AOM]; Multimedia Appendix 1). The videos were originally produced for the general English-speaking population. The participants were also asked to complete a short demographic survey.

### Sampling and Recruitment

Participants were eligible for the study if they were a practicing HCP serving Indigenous families in Alberta and could read and speak English. HCPs could be any health care personnel or professional interacting with Indigenous patients and families and providing care (eg, physician, nurse, or midwife). Participants were recruited from a variety of disciplines, including provincial health authorities and those working in not-for-profit organizations, local hospitals or care units, and Indigenous organizations, through a targeted recruitment effort using professional contacts and snowball sampling [22]. Purposive sampling was used to generate an in-depth understanding of the HCPs’ reflections [23]. Sample size was determined by evidence of data saturation, which was monitored through concurrent analysis of the data to assess the comprehensiveness, variation, and richness of the interviews [24].

### Ethics Approval

Ethics approval for this study (Pro00085188) was received by the appropriate Institutional Review Board offices at the University of Alberta. Before the interviews, all participants were informed of the study and the study’s purpose through recruitment emails during the screening for eligibility process and at the start of the interview sessions. All participants provided informed consent before any data were collected. The anonymity, confidentiality, and secure storage of the data were guaranteed to the participants in both the written consent form and before each interview and respected. A meal or electronic gift voucher to the value of CAD $25 (US $19) was provided to all the participants before commencing the interview in recognition of their time.

### Study Components

#### Demographic Questionnaire

The participants completed a demographic questionnaire via a secure web-based survey platform, REDCap (Research Electronic Data Capture; Vanderbilt University) [25]. The questionnaire asked the participants about their clinical or community health care practice history and setting, their Indigenous affiliation, and standard demographic information such as age and sex.

#### Semistructured Interview

Our research team initially developed the interview questions before field testing. The questions were then adapted over the course of 3 nonrecorded interviews with in-house volunteers (HCPs, researchers, and parents). The first part of the interview consisted of questions probing into the existing information sources and KT tools that were used by the HCPs and the patients they served. The second part of the interview consisted of questions specifically about the content and format of the croup and AOM videos. The semistructured nature of the interview schedule allowed flexibility for the interviewer (JK) to explore the most meaningful experiences for the participants. The participants were treated as experts of their own experience and had full autonomy over the information they shared.

Interviews were audio recorded and then transcribed verbatim by a third-party transcriptionist as data were collected. The transcripts were reviewed as the interviews were completed to ensure accurate interpretation and complete inclusion of the data collected in the interviews.

### Data Analysis

Data collection and analysis were conducted concurrently to facilitate a more focused and meaningful data collection. Data management and analysis were facilitated using NVivo software (version 12, QSR International). Transcripts were read several times, coded, and analyzed using thematic analysis to identify common themes across the interviews. The thematic analysis process followed was outlined by Braun and Clark [26] and entailed familiarization with the data, initial coding, grouping similar codes together, and the development of themes and subthemes. Themes were then named and described. The interviewer (JK) regularly debriefed the study team members SAE and LH. Iterative data collection and analysis continued until data saturation was reached.

A line-by-line approach was used during the coding process. An inductive approach was used where codes emerged from the data [27]. Data were coded and sorted based on the themes that developed throughout the analysis. A focused coding approach in which similar codes were grouped together was used to elucidate patterns during data analysis. Analytical rigor and trust were promoted through continual communication with the research team and a detailed study log. Interview recordings and detailed field notes promoted confirmability of the findings. Field notes promoted reflexivity, allowing for the acknowledgment of bias, transferability, dependability, credibility, and inductive research praxis [28].

A second researcher (SAE or KSW) re-examined the transcripts and the codes to mitigate interpretive bias. All concerns were discussed until a consensus was reached.

The description and interpretation of themes were then reviewed by a fourth research team member who self-identified as Indigenous (SDL) to ensure that the language, meaning, and context of the findings were appropriately reflective of Indigenous cultures and the needs faced by Indigenous families within the scope of this study.

## Results

### Study Participants

A total of 18 HCPs from various health professions (eg, physician, registered nurse, and licensed practical nurse) participated in the study. Participant demographics are presented in Table 1. All the participants were HCPs who served Indigenous families or communities; 83% (15/18) of the participants self-identified as female, and 39% (7/18) of the participants self-identified as Indigenous. The HCPs served Indigenous patients in rural communities (4/18, 22%), urban communities (6/18, 33%), or a mixture of both (8/18, 44%). The participants had, on average, 11 (SD 9) years of experience working with Indigenous communities.

**Table 1:** Demographic characteristics of the study participants (N=18).

Variable		Participants, n (%)
**Sex**		
	Male	3 (17)
	Female	15 (83)
**Age (years)**		
	21-30	5 (28)
	31-40	3 (17)
	41-50	3 (17)
	51-60	4 (22)
	31-70	3 (17)
**Indigenous descent**		
	First Nations	4 (22)
	Métis	3 (17)
	Inuit	0 (0)
	Non-Indigenous	11 (61)
**Level of education**		
	Bachelor of Arts (BA)	3 (17)
	Master of Science (MSc); Doctor of Philosophy (PhD)	2 (11)
	Nurse Practitioner (NP); Registered Nurse (RN)	5 (28)
	Doctor of Medicine (MD)	8 (44)
**Practice setting**		
	Private or community clinic	5 (28)
	Hospital	8 (44)
	Mixed or other	5 (28)
**Locale**		
	Rural	4 (22)
	Urban	6 (33)
	Both	8 (44)
**Working with Indigenous populations (years)**		
	<5	7 (39)
	5-10	4 (22)
	10-20	2 (11)
	≥20	5 (28)

^a^BA: Bachelor of Arts.

^b^MSc: Master of Science.

^c^PhD: Doctor of Philosophy.

^d^NP: Nurse Practitioner.

^e^RN: Registered Nurse.

^f^MD: Doctor of Medicine.

### Considerations for Adapting and Developing KT Tools for Indigenous Communities

The participants spoke about various considerations needed during the development or adaptation of KT tools to ensure they were relevant to the Indigenous communities they served. In total, 4 main themes were identified, and a summary of these themes can be found in Table 2. The participants described how addressing (1) accessibility, (2) relatability, (3) KT design, and (4) relationship building could produce more effective, culturally relevant, and meaningful KT tools.

**Table 2:** Descriptions and quotes of the identified themes and subthemes.

Themes and subthemes		Descriptions	Theme exemplars
**Accessibility**			
	Tangible resources Relationships	Tangible resources refer to the physical resources, or a lack thereof, that a caregiver or community has access to. This can include access to the internet, clean water, medication, medical devices, and transportation. Relationships refer to having consistent or easy access to HCPs^a^, services, and community connections.	“...if you were looking at antibiotics and like try to look at an antibiotic that while maybe if they’re like extremely rural and in the bush and they don’t have access to a refrigerator like you wouldn’t want to give them something that had to be refrigerated, right, like trying to take into consideration those little the tiny details in regards to lifestyle, where they’re from, what kind of life they live I guess” “not a lot of people have Wi-Fi because we’re very rural” “...often times families don’t have thermometers at home” “...accessing healthcare isn’t as easy, especially if they don’t have a physician or a nurse in their community...” “You have to weigh the pros and cons about going [to seek care]. Is it that severe that I have to go and wake up a nurse in the middle of the night? I think talking to rural communities and seeing their perspective on things and getting like an idea from a kid about how they actually access the healthcare services”
**Relatability**			
	Environmental contexts Individual representation Culture	Environmental contexts refer to the need for relatable scenery and environments, such as depicting characters from a remote community. Individual representation refers to having characters that resemble the target audience in terms of appearance, attire, and customs. Culture refers to integrating cultural practices and dynamics into KT^b^ tools and ensuring that these tools are culturally sensitive.	“...tailoring it more towards Indigenous culture, different languages, and incorporating Indigenous art might be helpful…” “I thought was not very Indigenous-friendly based on the lifestyle and the settings that most First Nations and Métis and Inuit people live” “Wow, I thought the second video actually looked maybe more visually that people would be an Indigenous kind of people. Maybe that would be more relatable if the people looked more like you. The other piece on that is that you might have a grandmother or somebody involved in the picture, which might also make it a little bit more relatable...” “...a lot of people have like Indigenous bedding like so if you had a baby...it would be nice to have like a baby wrapped in a special blanket things like that that are like very obviously Indigenous” “Yeah, like a blanket on a wall. Like a lot of people have those really nice Pendleton blankets on the wall on their like office or like of their home”
**KT design**			
	Format Plain language Translations	Format refers to the medium of a KT tool and the benefits and drawbacks that come with it; for example, video versus paper-based resource. Plain language includes avoiding medical jargon, using clear and simple wording, using active voice, avoiding excessive text, and using lower reading levels. Translations are important for ensuring that Indigenous patients and communities that speak Indigenous languages are able to use KT tools.	“...the more concise and brief, the more easily received...” “Videos are very short and concise and like even with the new videos that are coming out with AHS in regards to emergency and like their kiddos at the hospital those are very clear and concise and are easy to kind of absorb and understand...” “I think there’s many resources that could be dwindled down on their information or make it more like clear and concise so it’s easy for them to kind of absorb and not feel frightened about the information...” “They should really be striving to make resources that are adapted to their patients if they can because not all parents are willing to read a 30-page document” “I definitely think that translating them into languages like Cree or Dené or Stoney if we’re talking all through Alberta not just Northern Alberta and Blackfoot all of the languages I think would be very, very valuable” “...a lot of the Elders, a lot of grandparents, a lot of the people that would be relied upon to have this information or who would best have this information are more comfortable in their Indigenous language. I mean, the younger people, definitely a lot of them are more comfortable”
**Relationship building**			
	Understanding community needs Consultations while adapting tools Spreading information	Relationship building with community members, Elders, and HCPs is necessary to understand community needs using a bottom-up approach. Consultations with Indigenous community stakeholders are needed throughout the development and adaptation of KT tools to ensure that their needs are appropriately met. Connections with Indigenous stakeholders, HCPs, and community members are vital for distributing KT tools and facilitating the uptake of health information.	“Definitely consulting in the community with Elders to learn about their values and their cultural practices, just being very aware of that” “...we really need their [Elders] perspective and understanding of their resources when we’re creating these things and advising them...” “...But you have to work with the community. It can’t be done at the community, it needs to be done with the communities” “...I’m connected to my First Nation’s Facebook page and it’s like by far the easiest way to disseminate like videos, educational videos...” “I thought like Facebook would probably be like the easiest option (for dissemination) as well as just connecting with the community nurses”

^a^HCP: health care provider.

^b^KT: knowledge translation.

### Accessibility

#### Overview

HCPs described how the accessibility of KT tools and the resources mentioned within the tools varied greatly within the Indigenous communities they serve. HCPs often spoke of tangible resources (physical resources) that a family or community may or may not have access to as well as the relationships needed to have a consistent or an easy access to health care resources.

#### Tangible Resources

HCPs discussed access to tangible resources as a barrier to viewing KT tools and relating to the content within each tool. Access to internet, equipment, and health centers were mentioned as barriers to Indigenous community members seeking health information. Often, KT tools are disseminated on the web, which may not reach those without adequate internet connection. An HCP described the situation as follows:

Not a lot of people have Wi-Fi because we’re very rural, and I’m not sure what the cost of Wi-Fi is out there but not very many people have it and there’s often people who don’t have phone data.

Participant_018

This lack of internet reliability presents a challenge for the dissemination of and access to web-based KT tools.

Along with access to web-based resources, HCPs described the limited access their patients and some community members had to health centers. The representation of resources within the tools themselves was also discussed. The participants described the resources depicted in KT tools to which Indigenous community members might not have access. An HCP explained as follows:

I put out a doctor’s post about hand washing and then the Facebook comments were like well, it’d be nice if we had something to wash our hands with because at that time there was no hand sanitizer in the stores and they just kind of expected us to have a stock to give out.

Participant_010

Another HCP mentioned the following:

I think often times families don’t have thermometers at home, but I also see the value in explaining the difference between a low fever and a high fever. I don’t know so maybe you could just say if you have a thermometer it’s useful to see if it’s a low fever or a high fever.

Participant_016

These relatively small components of KT tool could impose a barrier for the user in understanding important content or acting upon specific recommendations. Similarly, another HCP described access to transportation as an issue, “*...*the Inuit people I work with don’t have cars*.*” One of the participants later went on to say, “There’s a variety of things that [these KT tools] have made assumptions about parents, that doesn’t always fit” (participant_007). As this HCP mentioned, finding a good fit with the message and audience is important for translating relevant evidence to various communities.

#### Relationships

When searching for health information, HCPs voiced several difficulties their community members faced when finding and receiving appropriate health care services. It was often described as *“*weighing up the pros and cons about going to seek care*”* (participant_008). Several HCPs mentioned that knowledgeable and accessible health care workers were already overwhelmed within the communities, so patients might not want to further burden them with requests or feel that such requests would put a strain on the provider-patient relationship. An HCP described their patient’s difficult decision-making, “Is it that severe that I have to go and wake up a nurse in the middle of the night?” (participant_008). This notion of balancing the barriers and benefits of seeking care was mentioned as particularly difficult for those in rural communities where access to care is limited. An HCP also suggested that researchers and practitioners would better understand Indigenous health care needs by “talking to rural communities and seeing their perspective on things” (participant_005).

### Relatability

#### Overview

Beyond assessing the resources in a given community, HCPs discussed how adapting KT tools to demonstrate relatable environmental contexts, individual representations, and the culture of a community can improve the relevance of the tools for Indigenous community members.

#### Environmental Contexts

HCPs described how familiar scenery could provide a more relatable tool for the intended community. For some communities residing in terrains difficult to navigate, physicians do not just wait in a clinic to see their patients. An HCP described their situation around traveling to visit the patients as follows:

Very often especially even in the summer with the storms, flights are restricted or cancelled. So, the ability to get in and out and right now it’s being served by a group of three flying docs who have been kind of commuting up.

Participant_005

The importance of tailoring visuals for increased relatability within the given community was also highlighted through the interviews. Upon viewing one of the KT tools, an HCP said the following:

I thought it was not very Indigenous-friendly based on the lifestyle and the settings that most First Nations and Métis and Inuit people live, it’s more middle class.

Participant_013

Furthermore, they explained the importance of representing the community setting for an individual to relate the content to their own experience. An HCP mentioned that this was perhaps why information was disseminated more broadly in a recent health campaign on social media as follows:

Yeah, or like I said, Indigenize it up. I know pretty clearly on my Facebook because I’m Cree, when Alberta Health Services posted that poster translated in Cree for washing hands, I saw that a lot more of my own relatives and a lot more on my Facebook share that than the poster of wash your hands.

Participant_012

#### Individual Representation

Similarly, in terms of visual representation, HCPs described how animated characters in the KT tools should look similar to the target communities. A self-identified Indigenous HCP mentioned the following:

I do think having representative characters would be a good starting point. Skin tone and skin coloration...It would be more relatable if people looked more like you...

Participant_015

Including HCPs that resemble Indigenous community members was also mentioned as a way to foster comfort and inclusion. Others discussed considering family structures, inclusion of grandparents as primary caregivers, and representative clothing animations as key considerations for improving relatability.

#### Culture

The idea of representing culture was a major discussion item throughout the interviews. HCPs felt that to engage with Indigenous communities, these KT tools required visual representations of the culture and ways of being that are unique to Indigenous peoples. “Indigenize it up” was the advice given by an HCP. More specifically, other HCPs suggested including items such as a Métis sash, traditional medicines, beadwork, and artwork on the walls to improve the relatability of the video:

Of course, like braids would be great and then yeah, bedding. Like a lot of people have like Indigenous bedding like so if you had a baby like it would be nice to have like a baby wrapped in a special blanket things like that that are like very obviously Indigenous...like physicians or doctors or nurses they have those nice beaded lanyards that are nice. Those are just like some ideas how to indigenize the space.

Participant_011

Cultural considerations as a whole were suggested as essential in producing relatable material for any specific community, not just for Indigenous peoples.

### KT Tool Design

#### Overview

In addition to questions regarding content, the participants were encouraged to comment on the design features of the KT tools and were asked for their opinions on the format of the tools and their preferences. Although design preferences were mixed, HCPs suggested that those developing or adapting KT tools should consider the appropriateness of format, depth of language, and translation of language when catering to Indigenous communities.

#### Format

Rather than one specific format recommendation, HCPs suggested that understanding the accessibility barriers would better inform the development of KT tool formatting. An HCP said the following:

There’s often people that don’t have phone data so I think there is still a role for physical pamphlets and handouts.

Participant_018

HCPs also suggested that the best format for KT tools is dependent on the context of the target audience.

#### Plain Language

HCPs recognized that a subsection of those they work with does not have English or health literacy, which would enable them to understand the health information in these KT tool formats. An HCP voiced this by saying the following:

We get a lot of patients especially from up north where English is not their first language or they’re not comfortable with English and like in a conversation you can slow down, you can change your words to make them simpler or like cue in what they understand but you can’t do that in a video.

Participant_010

In addition, while discussing the use of various KT tools more broadly, more verbose documents were identified as a barrier to effectively communicating with patients and families:

Often times, they’re really text heavy, they’re written at a higher level of reading, and have a lot of medical jargon.

Participant_012

These sorts of documents would not successfully inform the target audience and may even isolate users; therefore, HCPs suggested using plain language and avoiding jargon when creating or adapting KT tools for use by Indigenous groups.

#### Translations

When considering language, the idea of translating the tools into Indigenous dialects was discussed. HCPs offered suggestions of “translating them into languages like Cree or Dené or Stoney” but recognized the multitude of Indigenous languages spoken across Canada. Once again, HCPs urged that tool development match the unique needs of the community first, whether that involves translating the tools into a specific language or making use of subtitles or syllabics.

### Relationship Building

#### Overview

The most commonly mentioned theme through these discussions was the importance of building trusting relationships with community members to provide relevant information. HCPs believed that understanding community needs, consulting with community members throughout the KT process, and disseminating information in meaningful ways would promote effective practice in developing and adapting KT tools for use by Indigenous communities.

#### Understanding Community Needs

HCPs voiced the need for relationship building in Indigenous communities to inform content development in KT efforts. Understanding resources, services, and health provider access as well as common health concerns would require conversations with community members, which HCPs mentioned as being essential for creating meaningful products. An HCP said the following:

You have to work with the community. It can’t be done at the community, it needs to be done with the communities.

Participant_006

This collaboration was suggested not only for assessing needs but also for the continued adaptation and revision of KT tools.

#### Consultations

HCPs suggested that while adapting tools by fostering relationships with Indigenous community members, those facilitating KT efforts could seek relevant thoughts and opinions and adjust their products accordingly. Rather than simply improving the quality, an HCP said that community collaboration is a necessary component of KT efforts:

I think we really need their perspective and understanding of their resources when we’re creating these things and advising them.

Participant_009

#### Spreading Information

HCPs said that by understanding context, resources, and overall culture, one could plan an appropriate method of delivering health information to each community. An HCP recommended using social media to spread information:

I’m connected to my First Nation’s Facebook page and it’s by far the easiest way to disseminate educational videos.

Participant_003

Other HCPs had specific recommendations of where they have found success in dissemination but overall suggested consulting with community members throughout the KT process.

## Discussion

### Principal Findings

Cultural adaptation has the potential to enhance the acceptability, uptake, and adherence to evidence-based information. Where culture represents a dynamic set of norms, values, and practices in a social sphere, cultural adaptation focuses on modifying certain aspects to enhance the fit for an individual’s cultural values, preferences, and norms [29]. Incorporating Indigenous knowledge into KT efforts has the potential to create “new knowledge, policies, and practices to address health issues in communities” [30]. Unfortunately, KT has received relatively little attention in Indigenous health contexts.

As a starting point to understanding how KT tools could be adapted or tailored to meet the needs of Indigenous communities in Alberta, we assessed the value of 2 of our existing child health KT tools from the perspectives of HCPs. From our thematic analysis, 4 key themes were identified by HCPs to consider when adapting or developing child health KT tools use by for Indigenous families: accessibility, relatability, KT design, and relationship building. Here, we present important considerations for researchers and health professionals developing or adapting KT tools for use by Indigenous populations.

### Resources

Tangible resources, such as access to internet, being able to afford certain medications, or having access to clean water or transportation, were key elements to consider when portraying an event or message within the KT tools. In general, remote and rural communities have greater accessibility barriers than their urban counterparts and often face challenges in relation to transportation infrastructure and internet connectivity [31-34]. In addition, a community’s ability to leverage resources, services, and providers affects the relevance and applicability of the recommendations made in a KT tool. Researchers and developers should ensure that KT tools are tailored to the intended population or community with specific accessibility barriers in mind so that the users can implement the recommendations effectively.

Accessibility barriers can also impact whether users are able to access or view KT tools. For instance, caregivers residing in remote locales with sparse internet connectivity may be unable to use web-based KT tools. Although these interviews did not reveal a preference for a particular KT format, the ideal KT format is something which is easily accessed and used by the target population. In this case, social media platforms such as Facebook were identified as an extremely effective method for distributing web-based tools to populations with internet access and have been successfully used previously to share health information with First Nations communities within our province [35].

### Language

To ensure widespread accessibility and usability, the translation of KT tools into various Indigenous languages and dialects for specific communities and linguistic groups should be considered. However, a deep linguistic approach, as referred to by Resnicow et al [36], should be used. This involves ensuring that texts are culturally relevant rather than simply providing a direct translation. Direct language translations can often lack cultural nuances; therefore, high quality translations by Indigenous translators are needed.

A community-level collaboration approach should be adopted to support the development of community-owned products that incorporate cultural sensitivity and unique linguistic needs to improve their uptake and use within the community.

### Cultural Awareness and Understanding Diversity

Incorporating different aspects of a group’s culture *appropriately* into KT tools can foster engagement and increase knowledge, especially if shared through locally developed and contextualized ways [17,18]. Incorporating Indigenous cultural components, such as the medicine wheel, or practices, such as smudging, may help users to better connect with KT tools. An important caveat here is that Indigenous peoples and cultures are incredibly diverse [37], and as such, it is extremely challenging to create content that is relevant to all Indigenous groups within a single tool. However, culture has been successfully incorporated within KT efforts in the past. For instance, Laird et al [38] used a culturally safe KT approach to help Indigenous Australian caregivers manage protracted bacterial bronchitis in their children. Another study aimed to improve dementia health literacy in Canadian Indigenous communities by developing culturally appropriate fact sheets in collaboration with Indigenous organizations and Elders [39]. However, each Indigenous community has a unique culture and history; therefore, a successful approach to KT for one community may not be relevant to another. By actively engaging the community of interest, stakeholders could shape the information design and content to suit the community’s needs and preferences.

Ensuring that KT tools, specifically those that use visuals or graphics, incorporate relevant and appropriate depictions of environments and characters is important. Scenic or environmental portrayals must be relatable to KT users. Likewise, portrayals of characters within visual or story-based KT tools must also bear resemblance to the target users for optimal reception. This could involve having characters in a visual KT tool that have a similar skin complexion to that of the target audience and similar clothing items based on cultural or environmental factors or integrating customs and practices specific to that group [40,41].

Notably, those developing KT tools should avoid stereotypical or inaccurate representations of Indigenous peoples or cultures by effectively engaging with and involving the communities and tailoring the tools appropriately.

### Importance of Relationships

Relationship building is important throughout all aspects of the KT and dissemination process and is integral for working with Indigenous communities in a respectful and meaningful way.

Engaging with and building long-lasting mutual relationships with Indigenous communities, stakeholders, and Elders has been shown to be extremely important for working with Indigenous populations in a multitude of capacities and contexts [42-44]. A scoping review highlighted the importance of the contributions of the Indigenous Elders to well-being at the individual and community levels and the need to include Indigenous Elders in consultation and relationship-building efforts [45].

Relationship building entails creating long-term, sustainable partnerships with community members, stakeholders, and health professionals. Understanding community needs is required from a holistic and grassroots approach, which should only be achieved through genuine reciprocal relationships. Relationship building is also necessary to facilitate consultations with Indigenous community members, Elders, and health providers to adapt KT tools for use by their respective communities. KT tools should be codeveloped with the members of a given community to ensure that they are culturally sensitive, relevant to their needs, and accessible.

### Limitations and Future Directions

While reflecting on the enduring power dynamics of researchers engaging with Indigenous peoples [46], we believed that recruiting Indigenous community members required a sensitive process of meaningful reciprocity that was not feasible for this study. In consultation with Indigenous community representatives, it was suggested that HCPs who work with Indigenous families were well situated to comment on the available health care resources for the Indigenous communities they serve. The interviewed HCPs also provided important considerations on how to engage Indigenous patients and communities to develop relevant and impactful KT tools.

An important limitation to address is the biases that may arise from the type of qualitative research conducted in this study. Specifically, interpretive biases could arise during the interview or the data analysis and interpretation stages. To mitigate the impact of any interpretive biases, a second researcher (KSW) reviewed the data and brought forth any concerns for discussion with the primary author to reach a resolution. The findings of this study were also reviewed by one of our team members (SDL) who self-identified as Indigenous, further mitigating any interpretative biases.

Despite having a good representation from HCPs working directly with Indigenous families and communities, only 39% (7/18) of our participants self-identified as Indigenous. Even though the themes present across the interviews were similar, it is important to keep in mind that the lived experiences of HCPs who self-identify as Indigenous are distinct from those that are not. Future studies may benefit from incorporating a higher proportion of HCPs self-identifying as Indigenous or aim to recruit exclusively Indigenous participants. This may prove difficult given that Indigenous peoples have historically been underrepresented in health care–related fields [47]. It is important to recognize that given the small number of Indigenous people involved in the study, the findings are not generalizable to the broader population of Indigenous people. In addition, these findings only represent the perspectives of the HCP, and future studies should expand beyond consulting only HCPs and include the health consumers or knowledge users of the KT tool (Indigenous families). We recognize that this study is just a first step; consultations with Indigenous community members, Elders, Indigenous leaders, and other stakeholders are needed to fully understand how to best develop and adapt KT tools for use by Indigenous communities in accordance with their unique needs. Furthermore, we recognize that this study was conducted within a local context in Canada, and the findings might not be representative of all Indigenous populations, not just within Alberta but across Canada (and elsewhere). However, the croup video usability was completed at the Stanton Territorial Hospital in Yellowknife, Northwest Territories, Canada, which has a significant population of Dené people; Portage La Prairie, Manitoba, Canada, which has a significant population of Anishinaabe or Ojibwe, Cree, and Dakota or Sioux people; and the Stollery Children’s Hospital (Edmonton, Alberta, Canada). The AOM video usability was conducted in 3 rural emergency departments in Nova Scotia; the Royal University Hospital in Saskatoon, Saskatchewan, Canada; and Leduc Community Hospital, Alberta, Canada. Therefore, it is important to acknowledge that the perspectives of various Indigenous communities and Indigenous peoples may differ from the themes we have derived in this study. Future KT tool development studies may also benefit from usability testing that occurs across an increased number of locales, particularly those with significant populations of Indigenous peoples.

### Conclusions

Developing or adapting KT products that are relevant to different cultural groups is important to enhance the reach of knowledge and ultimately improve health outcomes. On the basis of the interviews with HCPs, accessibility, relatability, KT design, and relationship building were perceived by HCPs as important considerations for adapting and developing KT tools that would meet the needs of the Indigenous communities they serve. This study is a critical first step in identifying how to adapt existing KT tools for use by culturally and linguistically diverse communities in general. This study demonstrated the importance of engaging end users (health care consumers) in the development and critique of KT products. Including Indigenous perspectives in the continuing refinement of KT tool content and delivery will increase the relevance and accessibility of the messaging for future KT efforts in this unique population.

## Appendix

Multimedia Appendix 1 Examples of knowledge translation tools used.

## Abbreviations

AOM: acute otitis media
HCP: health care provider
KT: knowledge translation
REDCap: Research Electronic Data Capture

## Data Availability

These are available upon reasonable request from the corresponding author.
