# Supplementary material for: Adapting Child Health Knowledge Translation Tools for Use by Indigenous Communities: Qualitative Study Exploring Health Care Providers’ Perspectives
Source: JMIR Form Res. 2022 Oct 5;6(10):e36353. doi: 10.2196/36353 (PMC9582909; doi:10.2196/36353)
Supplement: Multimedia Appendix 1 [file formative_v6i10e36353_app1.pdf]

Multimedia Appendix 1

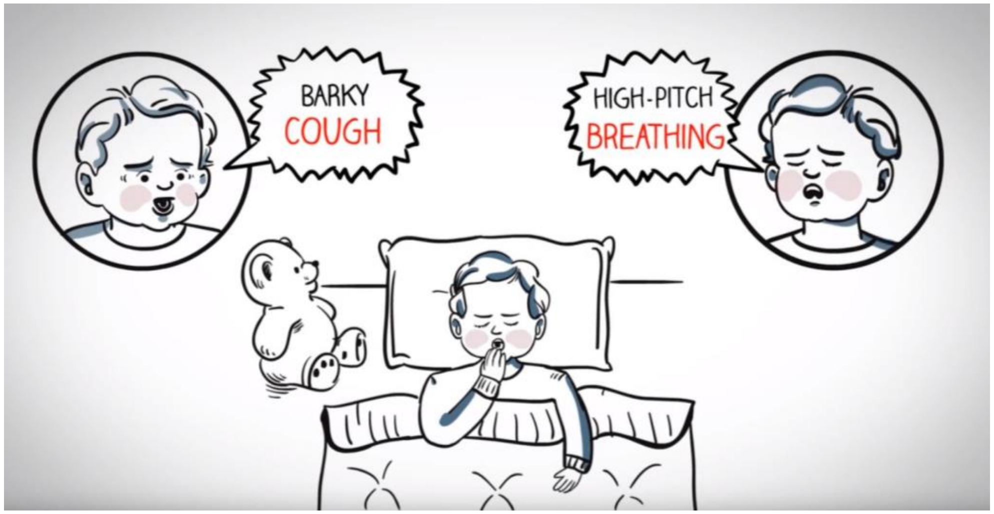

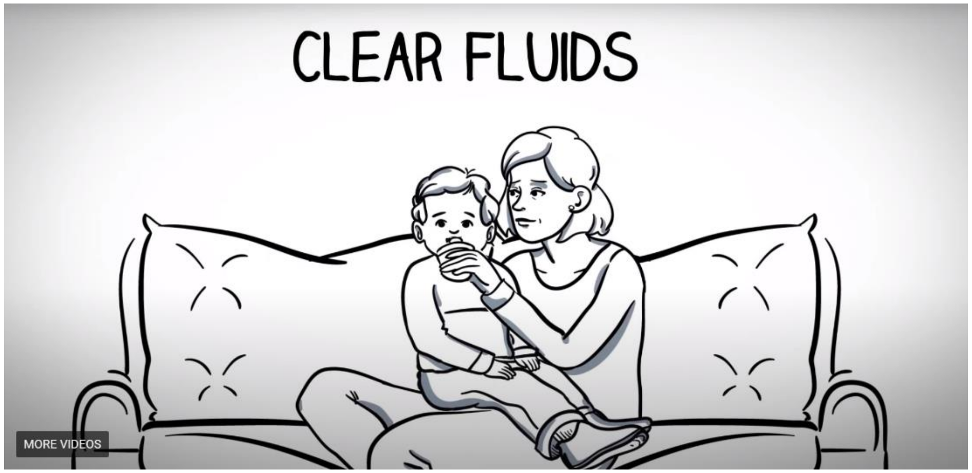

Example of Croup Whiteboard Animation Video

Available from: https://www.echokt.ca/tools/croup/

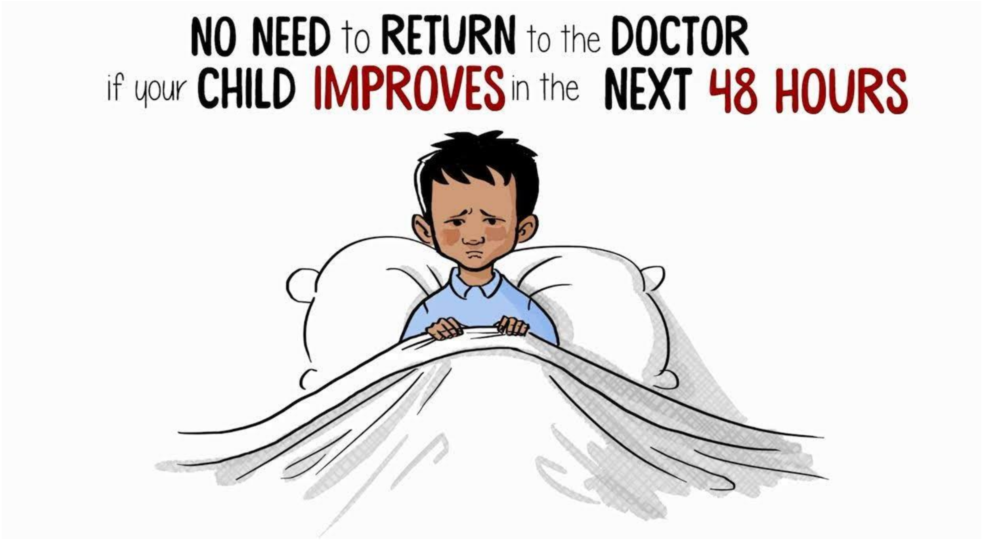

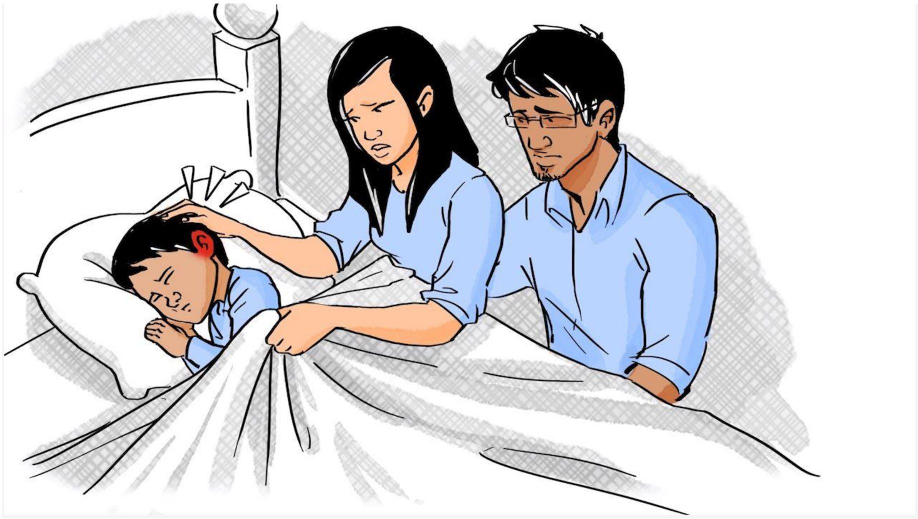

Example of Acute Otitis Media Whiteboard Animation Video

Available from: https://www.echokt.ca/ear-infection/
